# The Preliminary Study for the Trial of Using Selected miRNAs for the Laboratory Diagnosis of Colorectal Cancer

**DOI:** 10.3390/ijms27083702

**Published:** 2026-04-21

**Authors:** Michal Sulikowski, Tadeusz Sulikowski, Mateusz Kurzawski, Damian Malinowski, Elżbieta Urasińska, Monika Rac

**Affiliations:** 1 Department of General, Mini-Invasive and Gastroenterological Surgery Clinic, Pomeranian Medical University, Unii Lubelskiej 1, 71-252 Szczecin, Poland; 74301@student.pum.edu.pl (M.S.); tadeusz.sulikowski@pum.edu.pl (T.S.); 2 Department of Experimental and Clinical Pharmacology, Pomeranian Medical University in Szczecin, Powstancow Wielkopolskich 72, 70-111 Szczecin, Poland; mateusz.kurzawski@pum.edu.pl; 3 Department of Pharmacokinetics and Therapeutic Drug Monitoring, Pomeranian Medical University, Powstancow Wielkopolskich 72, 70-111 Szczecin, Poland; damian.malinowski@pum.edu.pl; 4 Department of Pathology, Pomeranian Medical University, Unii Lubelskiej 1, 71-252 Szczecin, Poland; elzbieta.urasinska@pum.edu.pl; 5 Department of Biochemistry, Pomeranian Medical University, Powstancow Wielkopolskich 72, 70-111 Szczecin, Poland

**Keywords:** miRNAs, colorectal cancer, genetic biomarkers

## Abstract

Colorectal cancer (CRC) is the second most common cancer in Europe. There is a need to explore and validate new blood-based tumor markers to improve the selection of patients who are likely to benefit from an early, non-invasive diagnosis of CRC. The purpose of this report is to present the test protocol and its verification. The study is planned in four phases. The first trial phase involves collecting material, consisting of healthy tissue, diseased tissue, and plasma, from 120 CRC patients over 50 years old during surgery. This phase also involves identifying microRNAs (miRNAs) and comparing their expressed levels in colorectal cancer cells with those in healthy tissue taken from a standard resection margin. We detected measurable levels of miRNAs in tissue samples taken from patients, confirming that the material was correctly removed for testing. Statistically significant differences were obtained between healthy and cancerous tissue for selected miRNAs. Some of the selected miRNAs have higher expression levels in CRC tissue and could be potential candidate biomarkers for laboratory-based colorectal cancer diagnosis.

## 1. Introduction

Colorectal cancer (CRC) is the second most common cancer, comprising approximately 13% of all new cancer diagnoses in Europe. Approximately 40–50% will develop metastases during the course of their disease [1]. Due to the high incidence of liver metastasis, CRC has a poor prognosis and represents one of the leading causes of cancer-related deaths worldwide [2]. Therefore, despite the improvements in diagnosis and treatment, research in this field remains of great interest.

The mechanisms and phenotypes of genetic alterations in colorectal tumors are diverse [3,4,5]. The chromosomal instability phenotype represents the most common group, characterized by frequent losses in chromosomal regions such as *17p, 18q, 5q, 8p*, and *20q*, as well as mutations in *TP53* or *KRAS*. The tumor is more frequently located in the distal colon. The second group is associated with genetic instability, such as the microsatellite high-instability (MSI-H) phenotype, due to a mismatch repair deficiency. This tumor phenotype is more frequently observed in the proximal part of the colon and in older women. The third group is associated with a *CpG* island methylation phenotype.

Although surgery remains the mainstay of curative treatment for CRC, many patients still have a high risk of disease recurrence. Therefore, it is imperative to identify prognostic markers that can help predict the clinical outcome of CRC. MicroRNAs (miRNAs) regulate approximately 30% of human genes [6] and show great potential as diagnostic and prognostic biomarkers for CRC. MiRNAs play crucial roles in various key cellular biological processes, including differentiation, proliferation, growth, migration, survival, and cell response to anticancer treatments [7]. Furthermore, it has been discovered that miRNA expression underscores their importance from both a basic science and a clinical perspective.

MiRNAs are highly conserved, 22-nucleotide, single-stranded RNAs. MiRNAs act as trans-acting factors of target genes, either suppressing protein translation or inducing mRNA degradation [6]. Non-coding miRNAs suppress gene expression by partially pairing with the 3′ untranslated region (3′-UTR) of targeted mRNAs [8]. Single-nucleotide polymorphisms (SNPs) in miRNA genes have been analyzed extensively for functional implications. A single base pair change in the nucleoside sequence can affect miRNA biogenesis, processing, and target site binding. As gene regulators, miRNAs are implicated in all cancer types studied. They are oncogenes or tumor suppressors [9]. This effect is underlined by amplification, deletion, and point mutations in miRNA loci, deregulation of transcription factors (which modulate of miRNA expression), epigenetic silencing, and inhibition of the processing of primary miRNA to its mature form. When miRNAs are upregulated, they can suppress genes involved in cell growth and proliferation or downregulate other miRNAs, thereby promoting the development or progression of cancer [10]. Depending on their role in the development of cancer, microRNAs (miRNAs) are classified as oncogenes, which activate oncogenesis or inhibit the expression of tumor suppressor genes (‘oncomiRNAs’), or tumor suppressors, which inhibit the expression of oncogenes or lead to meta-cell apoptosis (‘oncosuppressor miRNAs’) [11]. Indeed, dysregulation of the particles occurs in almost all humans with cancer. MiRNA-encoding genes are highly conserved and the high frequency of SNPs within these genes supports their functional importance [12]. Growing evidence also supports a link between SNPs in miRNA genes and CRC risk, prognosis, and drug response [13,14,15]. Therefore, miRNAs could be unique therapeutic targets in CRC [16].

The mechanisms underlying dysregulated miRNA expression in human cancers remain poorly understood. Overall, it is necessary to explore and validate new biomarkers to improve the selection of patients likely to benefit from early, non-invasive CRC diagnosis. The results of many studies are heterogeneous between trials and appear to be modulated by the chemotherapy regimen. Heterogeneity of results may also be influenced by tumor location [17]. Some miRNAs have conflicting results regarding their expression and regulation in tumor tissue, especially before and after chemotherapy. However, certain signaling pathways have been identified. For example, lethal-7 (let-7, including let-7a-1 miRNA) is a tumor-suppressor miRNA family that acts by down-regulating oncogenes, including *KRAS* [18,19]. Similarly, miR-128 is considered a tumor suppressor in CRC, inhibiting tumor growth and metastasis and sensitizing CRC cells to induce apoptosis by targeting the SIRT1/ROS/DR5 pathway [20]. MiRNA-143 has also been described to be downregulated in CRC, thereby altering RAS signaling and inhibiting tumor cell growth [21]. MiRNA-34a-5p is downregulated in CRC tumor tissues and induces cell apoptosis, cell cycle arrest at G1 phase and p53 transcriptional activity [22]. On the other hand, the miRNA-200 family (miRNA-200b, miRNA-200c, miRNA-141 and miRNA-429) is significantly overexpressed in CRC. The methylation process in CRC differs from that in healthy tissue. Specifically, demethylation occurs, leading to increased *miRNAs* genes expression of their promoter regions and promoting the transformation into cancerous tissue. Its expression is a potential biomarker for predicting lymph node metastasis and CRC recurrence [23,24]. A significant limitation in detecting miRNAs in archived tumor tissues is the heterogeneity within the same primary tumor and between different metastatic sites [25]. Therefore, evaluation of circulating miRNA expression in serum or plasma may be preferable for prognosis prediction in the clinical setting.

Blood-based tumor markers are gaining acceptance as a non-invasive way to detect cancer. They may be clinically valuable indicators of patient outcomes and the existing TNM staging system (T-Tumor, N-Nodes, M-Metastasis). MiRNAs are present in the human circulation in remarkably stable forms protected from endogenous ribonuclease activity [26]. As highly specific biomarkers, plasma miRNA-based assays may provide accurate methods for diagnosing and prognosing CRC [27,28,29,30]. Tsukamoto et al. reported that plasma exosomal miRNA-21 levels are a useful biomarker for predicting recurrence and poor prognosis in CRC [31]. Plasma miRNA-139-5p has been described as a biomarker for tumor recurrence and metastasis in CRC [32]. The miRNAs panel: let-7g, miRNA-21, miRNA-31, miRNA-92a, miRNA-181b and miRNA-203 was reported as a prognostic marker for CRC with 91% specificity and 93% sensitivity compared to the traditional markers (CEA and CA19-9). Evaluation of miRNA-21 expression showed that it discriminated between adenoma and CRC from healthy controls. Plasma miRNA-141 detection has been used as a complementary prognostic marker to CEA for detecting CRC patients with distant metastases [33].

So far, almost 100 miRNAs have been found to be up- or downregulated in CRC cells compared to healthy cells. The upregulated miRNAs are more frequently associated with chromosomal regions showing an increase in copy number, and the downregulated miRNAs are associated with deleted chromosomal regions. Table 1 shows up- or downregulated miRNAs in CRC cells compared to healthy cells and the miRNAs detected in the plasma of CRC patients.

It is important to continue research into miRNAs to understand their mechanism of action fully and to improve diagnosis and targeted cancer therapies in the future. Therefore, we would like to present our proposed protocol for the trial we are starting in our clinic. First, we will analyze samples from CRC tumors, healthy tissues, and blood plasma to identify candidate miRNAs associated with CRC presence and stage. This analysis will compare miRNA expression levels and their impact on target protein expression. In the second phase, we will validate the clinical significance of selected miRNAs as potential non-invasive biomarkers for predicting CRC presence, metastasis, tumor recurrence, and CRC patient prognosis. Based on the collected data, we will evaluate other potential predictive markers, including molecular ones. Using this systematic approach, we aim to demonstrate that plasma miRNA levels can serve as the clinical biomarkers. This study aims to identify a plasma-based CRC-specific predictive factor that correlates with miRNA expression in cancer tissue. Finally, we plan to develop a non-invasive, sensitive, and specific diagnostic test for CRC. The study is an independent and non-profit investigator-initiated trial. This brief report presents the preliminary results of this study, which compared healthy tissue with CRC tissue.

## 2. Results

The first group of patients have undergone surgery, and further participants are being recruited. Measurable levels of miRNA were detected in tissue samples taken from the patients. This confirms that the material was correctly removed for testing. Preliminary results on miRNA expression levels are presented in Table 2. Expression levels were measured as RQ (Relative Quantification) in two samples from one site of healthy tissue and two samples from one site of CRC tissue. The median miRBase ID-H is a geometric mean from RQ values of miRBase ID-H1 and RQ of miRBase ID-H2 (two samples from one area of healthy tissue). The median miRBase ID-C is a geometric mean from RQ values of miRBase ID-C1 and RQ of miRBase ID-C2 (two samples from one area of CRC tissue). These data were then used for the Wilcoxon signed-rank test. The results of this test are presented in Table 2 as *p*-values. Statistically significant differences were obtained between healthy and cancerous tissue for 7 of the 12 selected miRNAs. The most promising results concern: miRNA-181b-5p, miRNA-21-5p, miRNA-221-3p, miRNA-222-3p, and miR-93-5p. Unfortunately, miR-23a-5p is detectable in only certain patients in both healthy and diseased tissue. This may be related to its very low stability.

The relative expression is presented in Table 3. It was calculated as miRBase ID-C divided by miRBase ID-H.

## 3. Discussion

The best-performing test is a unique biomarker panel of a few miRNAs with high sensitivity (around 95%) and specificity (around 90%) for identifying cancerous lesions. Many different blood-based biomarkers have been investigated to detect advanced precancerous lesions, but few have progressed beyond the discovery stage. Some CRC biomarkers have been reported [37] to have high sensitivity and specificity, but more extensive prospective studies in unbiased screening populations are needed to validate them. For example, Li et al. performed [38] a meta-analysis of 10 studies from scientific databases to evaluate the diagnostic role of miRNA-21 in CRC samples and surrounding tissues. Potential target genes for miRNA-21 were predicted and evaluated using functional analysis. The data showed that the *miRNA-21* gene was different in colorectal cancer tissues and adjacent tissues and was an up-regulated gene. The molecular function focused mainly on cytokine receptor binding, and the biological process focused mainly on ubiquitin-dependent protein degradation mediated by the proteasome. The target genes were mainly distributed in tumor pathways. Another meta-analysis [39] evaluated the clinical utility of miRNA panels as potential biomarkers for diagnosing CRC. Subgroup and meta-regression analyses showed that miRNA panels had the highest diagnostic accuracy in serum samples compared to other sample types. MiRNA-15b, miRNA-21 and miRNA-31 showed the best diagnostic accuracy for CRC, with sensitivities and specificities of 95% and 94%, respectively. In the subsequent meta-analysis [40], several miRNAs (including miRNA21) were found to be dysregulated and to discriminate patients with CRC from healthy controls. However, there was little consistency across included studies, making it challenging to identify specific miRNAs that were consistently validated. A strong association between low *miRNA-451* expression levels and CRC progression was also observed. The miRNA-451 family may serve as a potential biomarker for early colorectal cancer diagnosis [41]. Finally, Moloudizargari et al. [42] found that a high level of *miRNA-31* expression is associated with poor overall and progression-free survival. The results showed that biomarker panels performed better than the corresponding individual biomarkers in diagnostics [43]. On the other hand, the diagnostic accuracy of miRNA panels in colorectal cancer (CRC) remains inconsistent, and there is a lack of analysis to determine which miRNA panels can serve as robust biomarkers for CRC diagnosis. Blood-based screening may improve test sensitivity for CRC detection. Therefore, we have designed a brief protocol to identify miRNAs in two tissue types. Different studies have shown that all selected miRNAs are either sensitive markers or markers of poor prognosis. In our studies, we observe clear differences between the expression of selected miRNAs in healthy tissues and tissues with CRC for 7 of the 12 selected miRNAs. The most promising results concern: miRNA-181b-5p, miRNA-21-5p, miRNA-221-3p, miRNA-222-3p, and miRNA-93-5p. The aim of further research is to investigate the associations between selected miRNAs and their presence in plasma, as well as the associations between plasma miRNAs and clinical data. Another objective is to develop a research protocol and a practical diagnostic test for detecting CRC.

## 4. Materials and Methods

### 4.1. Research Phases Description

The trial is planned in four phases.

1. The study’s first phase is to identify miRNAs and compare their expression in colorectal cancer cells and healthy tissue obtained from a standard resection margin. In addition, the aim of further research is to investigate the associations between selected miRNAs and their presence in plasma. The patient’s clinical data will be collected, and the disease stage will be confirmed by histopathological examination. A multidisciplinary research team will collaborate on this phase, including a surgeon, pathologist, geneticist, and laboratory diagnostician. The next step is to investigate potential areas of promoter methylation and target gene expression and to test the understanding of miRNA interactions with clinical parameters. A comparison with existing blood markers will also be carried out. The endpoint of phase one is feasibility, which involves evaluating whether research material with detectable miRNA levels can be obtained during surgery and whether the methodology for detecting selected miRNAs is repeatable. It also involves identifying those miRNAs that meet the following criteria: present in tumor tissue and absent in healthy tissue, or vice versa.
2. The second phase of the study will follow the progression of the disease and the response to the treatment used, analyzing patient survival rates and outcomes over 5 years.
3. The third phase focuses on developing diagnostic and prognostic tests. This part of the trial will be conducted independently of the second phase.

Phases two and three are associated with a prognostic endpoint, which involves establishing the association between specific miRNAs and their presence or absence in tumor tissue and plasma, and between tumor stage and progression-free survival, or overall survival within five years of diagnosis.

4. The fourth phase is a prospective study to confirm the validity of the developed diagnostic tests in a new randomized group without a history of CRC. Endoscopic screening will be supported by a 5-year follow-up. To increase the power of the study, this group of patients will be selected from those with CRC risk factors. Participants for the control group will be selected from patients visiting the genetic and gastroenterology clinics. These individuals will meet all the study’s exclusion criteria and will not have been diagnosed with CRC. They will be classified as high-risk for CRC. Patients are referred to the genetics clinic based on their medical history, where they are tested for mutations in the *MLH1*, *MSH2*, and *MSH6* genes. They will then be placed under observation by the gastroenterology clinic. The project involves a five-year follow-up with disease onset or absence as the endpoints, and plasma testing only. This study serves as a control for the miRNAs identified in phases 2–3, which are present or absent in both plasma and diseased tissue but absent in healthy tissue. The project does not assume that miRNA testing will influence clinical pathways. Phase four’s primary diagnostic endpoint is to evaluate the predictive value, sensitivity, and specificity of the new diagnostic test for detecting early-stage colorectal cancer through genetic testing, i.e., the presence or absence of miRNA markers in the plasma of the control cohort. This will then be followed up for five years, with the endpoint being CRC diagnosis or no diagnosis. It is planned to patent the diagnostic/prognostic test.

**Figure 1:**
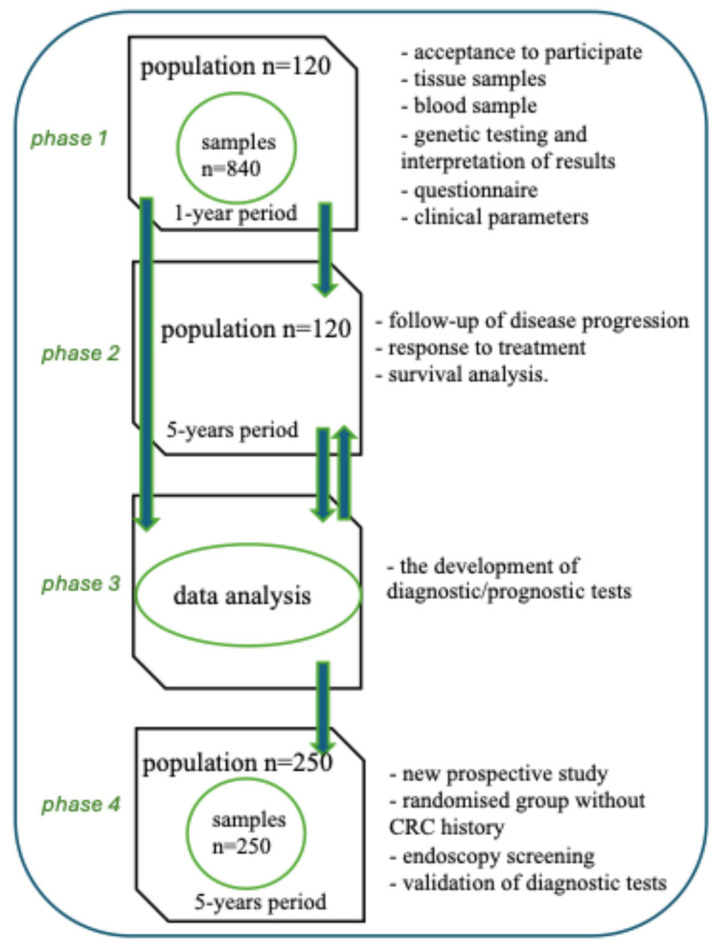
The description of the trial phases. ↑, ↓—the next step.

The description of the research phases is shown in Figure 1.

### 4.2. Study Group for First Part of the Trial

**Figure 2:**
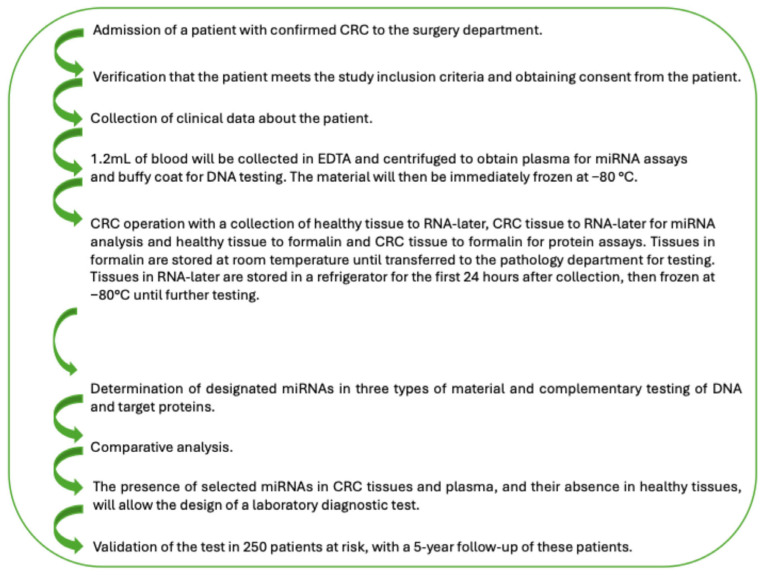
The study detailed procedure (phase 1).

The following study design will be used, as shown in Figure 2.

The study included 120 clinically stable patients with CRC aged 50 years or older. Patients who have previously received treatment are excluded to avoid potential metabolic changes induced by such treatments. The patients are all Polish residents treated in the Department of General, Mini-Invasive and Gastroenterological Surgery Clinic of Pomeranian Medical University in Szczecin (northwestern Poland) in 2024–2026. The study complied with the principles outlined in the Declaration of Helsinki and was approved by the institutional ethics committee at the Pomeranian Medical University (No. KB-006/27/2024). All subjects provided informed consent for inclusion in the study before participating.

The criteria for inclusion in the study:

- CRC eligible for surgery;
- Age over 50 years.

Exclusion criteria for patients in the study:

- Age under 50 years;
- History of other cancers;
- Receiving anti-cancer therapy (radio/chemotherapy);
- Concomitant colorectal disease other than CRC;
- History of autoimmune disease;
- Inability to take samples from healthy tissue due the small incision limit;
- History of carrying germline mutations in genes encoding repair proteins: *MLH1*, *MSH2*, and *MSH6*.

### 4.3. Blood and Tissue Samples

Fasting blood samples were collected for DNA extraction, and plasma samples were collected for miRNA expression measurements. Blood was collected from the patient before surgery and immediately centrifuged at 5800 rpm for 10 min. The plasma was then stored at −20 °C immediately after centrifugation. A few days later, the buffy coat and plasma samples were transferred safely to a freezer at −80 °C and stored until analysis.

We needed duplicate tissue sections from each patient (2 tubes with buffer and cancer tissue, 2 tubes with buffer and healthy tissue), i.e., 4 RNA-later tubes (Thermo Fisher Scientific Inc., Waltham, MA, USA) per person from 2 different collection sites. The RNA-later tubes were stored in a refrigerator at +4–8 °C for 24 h, then frozen and stored at −20 °C. A few days later, the tissue samples were transferred safely to a freezer at −80 °C and stored until assayed. The tissues were only defrosted once for testing. We needed tissue sections from each patient, collected in 2 formalin vials (1 formalin and tumor tissue, 1 formalin and healthy tissue), i.e., 2 formalin vials per person from 2 different collection sites. We stored the formalin collection tubes at room temperature until testing.

### 4.4. Colorectal Cancer Surgery

The surgical treatment of colorectal cancer is a comprehensive approach that involves the removal of the cancerous tumor along with the adjacent segment of the colon and regional lymph nodes. This advanced procedure is essential for complete excision of the malignant lesion and assessing disease staging. The surgeries can be performed using various techniques, ranging from minimally invasive methods, such as endoscopic tumor removal in very early cancer stages, to more advanced methods, such as laparotomy, laparoscopy, and surgeries assisted by robotic surgical systems. Traditional open surgeries are now usually reserved for large tumors with local infiltration of cancer into the tissues surrounding the colon. Before the surgery, the patient must undergo diagnostic tests to qualify for the procedure. The choice of surgical technique depends on the location and stage of the tumor. Before surgery, the patient must undergo appropriate preparation, including a low-residue, easily digestible diet and the use of laxatives before the procedure. Colon surgery usually begins with an incision in the right or left, or upper or lower midline of the abdomen, followed by an incision in the peritoneal layer, and in the next stage of the procedure, ligation of the artery supplying the relevant segment of the colon. The appropriate segment of the colon is then removed, and its lumen is closed at the proximal and distal ends. If an anastomosis cannot be performed, the colon stump may be brought to the surface to create a stoma.

Research material collected from healthy tissue was excised at a recommended distance of approximately 10 cm from the neoplastic lesion [44]. In our study, this distance was most commonly 12–14 cm. This approach increases the likelihood that the collected sample has not yet undergone advanced neoplastic processes. Furthermore, each collected specimen is submitted for histopathological examination to confirm the adequacy of tissue sampling and differentiate healthy tissue neoplastic tissue.

In our study, both neoplastic and healthy tissue samples are obtained from the same patient to ensure paired samples. The tissue samples therefore consist of four sets, each consisting of two tissue specimens of healthy and tumor tissue assigned to a specific individual patient.

### 4.5. Immunohistochemical Examination

Post-operative colorectal cancer specimens are sent to the Department of Pathology at the Pomeranian Medical University for routine microscopic examination. After fixation in buffered 10% formalin, the tissue is embedded in paraffin. For histopathological diagnosis, sections (4 μm thick) are stained with hematoxylin and eosin. In addition, one slide of cancerous tissue and one of healthy tissue are prepared for immunohistochemistry. Slides are deparaffinized, rehydrated, immersed in a pH 6.0 buffer, and heated in a water bath at 98 °C for 20 min to induce antigen retrieval. Endogenous peroxidase activity is then blocked. Slides are then incubated with primary antibody for 30 min, and immunostaining is performed using the Dako EnVision™+ Kit (Dako Denmark A/S, Glostrup, Denmark) according to the manufacturer’s instructions. The reaction is developed with a diaminobenzidine substrate chromogen solution, and the slides are counterstained with hematoxylin.

### 4.6. miRNAs Examination

All genetic testing is performed in the Department of Experimental and Clinical Pharmacology of Pomeranian Medical University.

Tissue fragments (both from tumor and healthy colon, ~50 mg) are dissected and immediately immersed in RNA-later (Thermo Fisher Scientific Inc., Waltham, MA, USA) to prevent RNA degradation, then stored at −80 °C until analysis. Total RNA (including small RNA) is extracted from tissue samples using a TRIzol-based kit, including treatment with DNase (Direct-zol RNA Miniprep Plus kit, Zymo Research, Irvine, CA, USA). Briefly, frozen samples (up to 50mg) were homogenized in 600 µL TRI reagent using rotor-stator homogenization method. Samples were centrifuged to remove debris and mixed with an equal volume of ethanol. The rest of the procedure involves passing samples through a Zymo-Spin™ column (Zymo Research, Irvine, CA, USA), washing, performing in-column DNase I treatment, and eluting RNA with ultra-pure water. Subsequently, RNA concentration and purity are measured using a UV spectrometer.

Reverse transcription (RT) is performed using 10 ng of total RNA and a kit including the preliminary steps of 3’ poly-A tailing and 5’ ligation of an adaptor sequence to allow synthesis of cDNA template in one reaction from all mature miRNAs with universal primers (TaqMan™ Advanced miRNA cDNA Synthesis Kit, (Thermo Fisher Scientific Inc., Waltham, MA, USA). Quantitative PCR is performed using a real-time PCR system (QuantStudio™ 7 Pro, Thermo Fisher Scientific Inc., Waltham, MA, USA), dedicated TaqMan™ Fast Advanced Master Mix, and TaqMan Advanced miRNA Assays (Thermo Fisher Scientific Inc., Waltham, MA, USA), with previously synthesized cDNA as a template. Amplification includes the selected miRNAs investigated in colorectal cancer and two miRNAs validated to serve as an endogenous control (each target in a separate reaction of 10 µL, 40 PCR cycles)—miRNA-186-5p and miRNA-361-5p. The endogenous controlled miRNAs were selected based on the literature [45,46,47]. The quantitative expression of individual miRNAs is calculated using the ΔCt method in relation to the mean expression of two endogenous controls. The obtained relative quantity values (RQ) can be further processed for statistical analysis. Table 4 lists the miRNAs finally selected for analysis. For the preliminary analysis, we selected miRNAs for which the presence in colorectal cancer tumor tissue and their potential association with diagnosis or progression have already been reported in the literature [15,25]. Furthermore, *PTEN* is the target gene for all the selected miRNAs. The selection also included those described in the literature as overexpressed or silenced, associated with a poorer prognosis for patients, or already being tested in clinical trials in vivo.

### 4.7. Statistical Analyses

Two samples of healthy tissue and two samples of diseased tissue were taken from the same location on each patient. The expression results for each patient are reported as RQ = 2^−ΔCT^. The geometric mean was then calculated for each patient from the two healthy tissue samples and the two diseased tissue samples. These data were then used for the Wilcoxon signed-rank test. Relative expression was calculated by dividing the geometric mean of the diseased tissue by the geometric mean of the healthy tissue. Due to the asymmetric distribution of the data, the descriptive statistics are presented using the median and the Q1 and Q3 quartiles. The above data are presented in Table 3 as a result of the preliminary study.

In subsequent steps, non-parametric tests will be used to investigate associations and correlations between expression and clinical data. Univariate analyses will be performed using Fisher’s exact test, the chi-squared test, and independent samples *t*-tests. In abnormal data distributions, associations between qualitative variables and miRNA expression will be tested using the Mann–Whitney U test, while correlations between miRNA expression and quantitative variables will be assessed using the Spearman rank correlation coefficient (Rs). Multivariate models will be fitted using generalized estimating equations to compare changes in each outcome by test. *p* values <0.05 were considered statistically significant. Additional analysis will be performed with Bonferroni correction for multiple comparisons to determine the actual level of statistical significance. In subsequent phases, ROC analysis and the Cox model will be performed for the specified endpoints.

### 4.8. Limitation of the Study

The lack of protocol standardization is a limitation of the current study. The methodology description should be treated as a brief report rather than a standardized study protocol. Currently, it is not possible to validate the individual stages of the project.

## 5. Conclusions

This report presented the test protocol and its verification. The measurable miRNA expression levels in tissue samples from patients confirm that the material was correctly removed for testing. Some of the selected microRNAs have higher expression in CRC tissue and could be a potential biomarker candidate for the laboratory diagnosis of colorectal cancer. Further validation of the study is required.

## Figures and Tables

**Table 1:** miRNAs regulated in CRC cells and miRNAs present in plasma of CRC patients (based on references [5,15,25,34,35,36]).

Expression	miRNA Present in Tumor Tissue	miRNA Present in Plasma of CRC Patients
downregulated	miRNA-1, miRNA-26, miRNA-30a, miRNA-30c *, miRNA-103, miRNA-125a, miRNA-125b, miRNA-126, miRNA-132 *, miRNA-133a, miRNA-137, miRNA-140, miRNA-143*, miRNA-145, miRNA-191, miRNA-192, miRNA-195 *, miRNA-214, miRNA-215, miRNA-299, miRNA-337, miRNA-342, miRNA-370, miRNA-497	miRNA-29c, miRNA-126 **, miRNA-148a, miRNA-149, miRNA-150, miRNA-194 **, miRNA-375, miRNA-486, miRNA-944, miRNA-1180, let-7 familly
upregulated	miRNA-7, miRNA-9, miRNA-16 *, miRNA-17a *, miRNA-18a, miRNA-19a, miRNA-19b, miRNA-20, miRNA-20a, miRNA-21 *, miRNA-23a *, miRNA-25, miRNA-27a *, miRNA-29a, miRNA-29b, miRNA-29c, miRNA-30b*, miRNA-30d *, miRNA-31 *, miRNA-32, miRNA-33a, miRNA-33b, miRNA-34a, miRNA-92a, miRNA-93, miRNA-95, miRNA-96, miRNA-101, miRNA-106a, miRNA-106b, miRNA-124 *, miRNA-130, miRNA-135a, miRNA-135b, miRNA-141, miRNA-146a *, miRNA-148a *, miRNA-150 *, miRNA-154, miRNA-181a *, miRNA-18b *, miRNA-182d, miRNA-183, miRNA-188, miRNA-196a *, miRNA-199b, miRNA-200a,b,c, miRNA-203 *, miRNA-205, miRNA-210 *, miRNA-218, miRNA-219, miRNA-222 *, miRNA-223 *, miRNA-224 *, miRNA-372, miRNA-378, miRNA-429, miRNA-494, miRNA-625, let-7 familly (let-7a, b, g)	miRNA-15b, miRNA-17 **, miRNA-18a, miRNA-18b, miRNA-19a, miRNA-20, miRNA-21, miRNA-23a,b, miRNA-25, miRNA-27a, miRNA-29a **, miRNA-30e, miRNA-34a, miRNA-92a **, miRNA-103a **, miRNA-127, miRNA-141 **, miRNA-146a, miRNA-151a, miRNA-155 **, miRNA-181a, miRNA-183, miRNA-200b,c, miRNA-203, miRNA-211, miRNA-218, miRNA-221 **, miRNA-223, miRNA-345, miRNA-425, miRNA-449a, miRNA-584, miRNA-618, miRNA-720 **, miRNA-762, miRNA-885, miRNA-1290, miRNA-141 + 200 panel **

* associated with a poor prognosis, ** higher sensitivity and specificity.

**Table 2:** The relative quantification of miRNAs expression in healthy (H) and CRC (C) tissues and the results of their pairing (*p*).

miRBase ID	Median	Q1	Q3	*p*
miR-106a-5p-H	0.179	0.092	0.242	0.826
miR-106a-5p-C	0.205	0.090	0.244	
miR-130b-5p-H	0.007	0.005	0.012	0.249
miR-130b-5p-C	0.021	0.009	0.024	
miR-181a-5p-H	1.62	1.11	1.76	0.013
miR-181a-5p-C	2.25	1.96	3.42	
miR-181b-5p-H	1.15	0.602	1.66	0.001
miR-181b-5p-C	1.89	1.24	2.58	
miR-203a-3p-H	0.149	0.145	0.327	0.056
miR-203a-3p-C	0.426	0.221	0.690	
miR-20a-5p-H	1.47	1.35	1.85	0.041
miR-20a-5p-C	2.76	2.26	5.07	
miR-21-5p-H	10.9	7.02	16.9	0.001
miR-21-5p-C	81.1	45.6	102	
miR-221-3p-H	3.31	2.26	7.39	0.002
miR-221-3p-C	6.63	3.40	15.4	
miR-222-3p-H	0.259	0.207	0.441	0.004
miR-222-3p-C	0.555	0.424	0.855	
miR-23a-5p-H	0.033	0.014	0.090	1.00 *
miR-23a-5p-C	0.041	0.030	0.160	
miR-23b-5p-H	0.003	0,003	0.006	0.432
miR-23b-5p-C	0.004	0.001	0.006	
miR-93-5p-H	0.872	0.696	0.969	0.002
miR-93-5p-C	1.65	1.37	2.89	

miRBase ID—microRNA identification database, H—healthy tissue, C—CRC tissue, Q1—lower quartile, Q3—upper quartile, * the result is not representative due to the limited amount of data available.

**Table 3:** The relative expression of selected miRNAs.

miRBase ID	Median	Q1	Q3
miR-106a-5p	1.08	0.434	3.12
miR-130b-5p	1.67	0.851	2.10
miR-181a-5p	1.77	1.08	2.14
miR-181b-5p	1.72	1.31	2.11
miR-203a-3p	2.43	1.43	3.24
miR-20a-5p	1.66	1.50	2.25
miR-21-5p	5.96	3.20	11.7
miR-221-3p	1.85	1.16	2.09
miR-222-3p	2.30	1.37	3.18
miR-23a-5p	1.43	0.305	4.84
miR-23b-5p	0.80	0.356	1.56
miR-93-5p	1.99	1.54	3.82

**Table 4:** The levels of miRNAs are determined in the plasma and tissues of CRC patients during the trial’s first phase.

miRBase ID	Target Gene	Chromosomal Location	References
miRNA-20a	*PTEN, TMP1*	13q31.3	[48]
miRNA-21	*PTEN, BCL2, PDCD4, TIMP3, SPRY2, SERPINB5, RECK, TIMP3, TIAM1*	17q23.1	[31,49,50,51,52,53]
miRNA-23a	*PTEN, TLR2, APAF1*	19p13.12	[52,54,55]
miRNA-23b	*TMEM127, PTEN, SMAD3, RUNX2, TFAM, Zeb1*	9q22.32	[54]
miRNA-93	*RBL2, BMP2, PTEN*	7q22.1	[56,57]
miRNA-106a	*PTEN, E2F1, RB1*	Xq26.2	[58,59]
miRNA-130b	*SIRT4, PTEN*	22q11.21	[60,61]
miRNA-181a,b	*PTEN, OPALIN CYC1, TNS3, E2F5 GATA6, PPP3CB, ELF5*	9q33.3	[62,63,64]
miRNA-203a	*PTEN, EMP1, SOX2, KLF4*	14q32.33	[49,65,66]
miRNA-221	*PTEN, PAK1, TIMP2, p27Kip1, TYRP1, Ang2*	Xp11.3	[29]
miRNA-222	*PTEN, BBC3, PDE3A*	Xp11.3	[67]

## Data Availability

The Department of Biochemistry at the Medical University of Pomerania will archive the complete study records of each patient. The original contributions presented in this study are included in the article. Further inquiries can be directed to the corresponding author.
